# Upfront surgery is not advantageous compared to more conservative treatments such as observation or medical treatment for patients with desmoid tumors

**DOI:** 10.1186/s12891-020-03897-9

**Published:** 2021-01-05

**Authors:** Shinji Tsukamoto, Piergiuseppe Tanzi, Andreas F. Mavrogenis, Manabu Akahane, Akira Kido, Yasuhito Tanaka, Marilena Cesari, Davide Maria Donati, Alessandra Longhi, Costantino Errani

**Affiliations:** 1 Department of Orthopaedic Surgery, Nara Medical University, 840, Shijo-cho, Kashihara-city, Nara 634-8521 Japan; 2 Department of Orthopaedic Oncology, IRCCS Istituto Ortopedico Rizzoli, Via Pupilli 1, 40136 Bologna, Italy; 3 First Department of Orthopaedics, National and Kapodistrian University of Athens, School of Medicine, 41 Ventouri Street, Holargos, 15562 Athens, Greece; 4 Department of Health and Welfare Services, National Institute of Public Health, 2-3-6 Minami, Wako-shi, Saitama 351-0197 Japan; 5 Department of Rehabilitation Medicine, Nara Medical University, 840 Shijo-cho, Kashihara-city, Nara 634-8521 Japan; 6 Department of Medical Oncology, IRCCS Istituto Ortopedico Rizzoli, Via Pupilli 1, 40136 Bologna, Italy

**Keywords:** Desmoid tumor, Aggressive fibromatosis, Active surveillance, Surgery, MSTS functional score

## Abstract

**Background:**

This study compared the clinical and functional outcomes of patients initially treated with observation or medical treatment with those of patients treated with local treatment (surgery alone or surgery with adjuvant radiotherapy) to confirm whether observation or medical treatment is an appropriate first-line management approach for patients with desmoid tumors.

**Methods:**

We retrospectively reviewed the medical records of 99 patients with histologically confirmed primary desmoid tumors treated between 1978 and 2018. The median follow-up period was 57 months. We evaluated event-free survival, defined as the time interval from the date of initial diagnosis to the date of specific change in treatment strategy or recurrence or the last follow-up.

**Results:**

An event (specific change in treatment strategy or recurrence) occurred in 28 patients (28.3%). No significant difference in event-free survival was found between the first-line observation/medical treatment and local treatment groups (*p* = 0.509). The median Musculoskeletal Tumor Society score of the patients treated with first-line local treatment was 29 (interquartile range [IQR], 23–30), whereas that of the patients managed with first-line observation or medical treatment was 21 (IQR, 19–29.5). First-line observation or medical treatment was more frequently chosen for larger tumors (*p* = 0.045). In the patients treated with local treatment, local recurrence was not related to the surgical margin (*p* = 0.976).

**Conclusion:**

Upfront surgery is not advantageous compared to more conservative treatments such as observation or medical treatment for patients with desmoid tumors.

**Supplementary Information:**

The online version contains supplementary material available at 10.1186/s12891-020-03897-9.

## Background

A desmoid tumor (DT) or aggressive fibromatosis is a “clonal fibroblastic proliferation that arises in the deep soft tissues and is characterized by infiltrative growth and a tendency toward local recurrence but an inability to metastasize even though it may be multifocal in the same limb or body part” [1]. Before the 2000s, surgery with microscopically negative margins was considered the standard of care for patients with DT, which is similar to the treatment approach for soft tissue sarcomas of the extremities owing to the infiltrative growth pattern of DT [2]. However, DT resection often leads to substantial function impairment and cosmetic alterations in patients affected by an otherwise benign disease, without avoiding the high risk of local recurrence despite the wide margins of resection [2]. For this reason, a shift toward more conservative management has been introduced recently [3]. In accordance with this approach, an observation alone strategy has been recommended as the first-line approach in newly diagnosed patients, reserving chemotherapy, a tyrosine kinase inhibitor, radiation, and surgical treatment for cases of disease progression [4]. According to the recent consensus recommendations from the Desmoid Tumor Working Group, management of asymptomatic patients with initial observation, independent of tumor site and size, can be proposed [5]. An earlier decision toward an active treatment is needed for DTs located close to critical structures (e. g., mesenteric or head and neck), and for sites other than the abdominal wall, medical treatment should be considered as the first treatment option in case of progression [5]. In addition, because DT is a benign and symptomatic tumor, studies focusing on patient outcomes in terms of symptoms and function are needed [6].

Our objective was to investigate whether observation or medical treatment is an appropriate first-line management approach for patients with DT. Therefore, we performed a retrospective study to compare the clinical and functional outcomes of initial observation or medical treatment, with initial local treatment (surgery with or without radiotherapy).

## Methods

We retrospectively reviewed the medical files of 253 patients with histologically confirmed DT who were treated at the senior author’s institution between 1978 and 2018. As intra-abdominal DT is a completely different disease from extraperitoneal DT with regard to various factors (associated with familial adenomatous polyposis, treatment strategy, and prognosis), patients with intra-abdominal DT were excluded from this study [7]. The management of DT has evolved over the decades. Before 2010, we treated DTs with surgery. Recently, we observed patients with newly diagnosed DT following current practice paradigms, that is, observation as the first-line approach. Once patients showed clinical progression or symptoms, we switched to local or medical treatment. We evaluated the patients’ characteristics, DT size, treatment approaches, surgical margins, and patient outcomes (Tables 1 and 2). Thirty-seven patients with < 12 months of follow-up, 113 patients with missing data, and 4 patients referred for tumor recurrence were excluded. The remaining 99 patients were included in this study for further analysis. The characteristics of the included and excluded patients are shown in the Appendix. The median follow-up period was 57 months (interquartile range [IQR], 33–86 months). As first-line management, local treatment was performed in 60 patients (45 patients received surgery alone, and 15 patients received surgery with adjuvant radiotherapy) and observation (16 patients) or medical treatment (16 patients received low-dose chemotherapy, and 7 patients received non-steroidal anti-inflammatory drugs [NSAIDs] alone or a combination of NSAIDs and anti-hormonal therapy [tamoxifen]) was chosen for 39 patients. All patient data were retrieved from the patients’ medical records. All the patients provided written informed consent for the inclusion of their data in this study. Institutional review board/ethics committee approval was not considered necessary for retrospective studies at the senior author’s institution.

**Table 1:** Details of the patients included in this study at baseline

Variables	Patients (***n*** = 99, %)	Initial treatment		***P*** value
		Local treatment (***n*** = 60, 60.6%)	Observation/medical treatment (***n*** = 39, 39.4%)	
***Age at diagnosis (years)***				
Median	38.2	39.4	36.1	0.670
IQR	25.4–47.7	25.5–50.8	23.8–44.3	
***Sex***				
Male	37, 37.4%	23, 38.3%	14, 35.9%	0.806
Female	62, 62.6%	37, 61.7%	25, 64.1%	
***Tumor site***				
Abdominal wall	4, 4.0%	2, 3.3%	2, 5.1%	0.901^c^
Upper extremity	10, 10.1%	9, 15.0%	1, 2.6%	
Lower extremity	39, 39.4%	21, 35.0%	18, 46.2%	
Girdle	31, 31.3%	20, 33.3%	11, 28.2%	
Head and neck	6, 6.1%	2, 3.3%	4, 10.3%	
Chest wall	9, 9.1%	6, 10.0%	3, 7.7%	
***Tumor size (cm)***				
Median	8	7	9	0.045^a^
IQR	5–11	5–10	6–13	
***Clinical presentation***				
Mass	42, 42.4%	23, 38.3%	19, 48.7%	0.308^d^
Pain	7, 7.1%	3, 5.0%	4, 10.3%	
Mass + Pain	46, 46.5%	33, 55.0%	13, 33.3%	
Functional impairment	2, 2.0%	0	2, 5.1%	
Functional impairment + Pain	2, 2.0%	1, 1.7%	1, 2.6%	
***Previous surgery or trauma at the site of the primary tumor***				
Yes	14, 14.1%	9, 15.0%	5, 12.8%	0.721^b^
No	85, 85.9%	51, 85.0%	34, 87.2%	
***Year of diagnosis***				
1999–2009	61, 61.6%	48, 80.0%	13, 33.3%	< 0.001^a^
2010–2018	38, 38.4%	12, 20.0%	26, 66.7%	

*IQR* interquartile range. ^a^The difference was significant. ^b^The Fisher exact test was used. ^c^Comparison of extremity and non-extremity. ^d^Comparison of mass alone and the others

**Table 2:** Treatment characteristics and outcome of the patients included in this series

Variable	Patients (***n*** = 99, %)	Initial treatment	
		Local treatment (***n*** = 60, 60.6%)	Observation/medical treatment (***n*** = 39, 39.4%)
***Biopsy***			
Core needle biopsy	70, 70.7%	38, 63.3%	32, 82.1%
Open biopsy	29, 29.3%	22, 36.7%	7, 17.9%
***Total number of surgeries***			
Median	1	1	0
IQR	0–1	1–2	0–0
***NSAIDs***			
No	82, 82.8%	60, 100.0%	22, 56.4%
Yes	17, 17.2%	0	17, 43.6%
***Tyrosine kinase inhibitor***			
No	98, 99.0%	60, 100.0%	38, 97.4%
Yes	1, 1.0%	0	1, 2.6%
***Anti-hormonal therapy + NSAIDs***			
No	87, 87.9%	59, 98.3%	28, 71.8%
Yes	12, 12.1%	1, 1.7%	11, 28.2%
***Low-dose chemotherapy***			
No	72, 72.7%	56, 93.3%	16, 41.0%
Yes	27, 27.3%	4, 6.7%	23, 59.0%
***Follow up period (months)***			
Median	57	58.5	55
IQR	33–86	35–96	27–85
***Event***			
No	71, 71.7%	41, 68.3%	30, 76.9%
Yes	28, 28.3%	19, 31.7%	9, 23.1%
***Interval between the diagnosis and event***			
Median	18	19	13
IQR	9–32	9–36	9–24
***MSTS score***			
Median	26	29	21
IQR	21–30	23–30	19–29.5

*IQR* interquartile range, *NSAIDs* non-steroidal anti-inflammatory drugs, *MSTS* Musculoskeletal Tumor Society

We analyzed the prognostic values of age at presentation, sex, DT site and size, previous surgery or trauma in the area of the primary tumor, biopsy technique, initial treatment, year of diagnosis, adjuvant radiotherapy, and surgical margin. We divided the patients into 2 groups according to the tumor site because tumors in the extremities were reported to be associated with a higher local recurrence rate [8].

DT size was defined as the greatest dimension on imaging before any treatment. Low-dose chemotherapy with methotrexate at a dose of 30 mg/m^2^ plus vinblastine at a dose of 6 mg/m^2^, as previously reported [9], was administered to 27 patients for a median period of 12 months (IQR, 6–15 months) in the overall therapeutic process. Six (22.2%) of the patients required repeated low-dose chemotherapy. NSAIDs were administered to 17 patients for a median period of 13 months (IQR, 4.5–25 months); tyrosine kinase inhibitor, to one patient for 12 months; and a combination of NSAIDs and anti-hormonal therapy, to 12 patients for a median period of 14 months (IQR, 4.5–22.5 months) in the overall therapeutic process.

Routine clinical and imaging follow-up evaluations were performed at 8 and 12 weeks, and then every 3 months for the first year, followed by every 6 months for the next 4 years and yearly thereafter. Response to medical treatment was assessed on the basis of the DT size reduction, along with computed tomography (CT) or magnetic resonance imaging (MRI) for the best response, and was rated as “complete response,” “partial response,” “stable disease,” or “progressive disease” according to the modified Response Evaluation Criteria in Solid Tumors (version 1.1), which assesses the tumor extent on the basis of the sum of the longest diameter of all target lesions [10]. Functional scores according to the Musculoskeletal Tumor Society (MSTS) scoring system were collected for patients with extremity DTs when possible [11]. The MSTS score was based on 3 general factors (pain, function, and emotional acceptance) and 3 lower limb factors (use of supports, ability to walk, and gait) [11].

The chi-square or Fisher exact test was used to evaluate the association between categorical variables, as appropriate. The differences between the independent continuous variables were statistically analyzed using the Mann-Whitney *U* test for nonparametric analyses. Event-free survival was defined as the time interval from the date of initial diagnosis to the date of specific change in treatment strategy or recurrence or the last follow-up. The specific change in treatment strategy was defined as the change from observation or NSAIDs/anti-hormonal therapy to low-dose chemotherapy or surgery, or the change from low-dose chemotherapy to surgery. Recurrence-free survival was defined as the time interval from the date of initial diagnosis to the date of recurrence or the last follow-up. Event- and recurrence-free survival were evaluated with the Kaplan-Meier survival analysis, and survival curves were compared using a log-rank test. A *p* value < 0.05 was considered statistically significant. Analyses were performed using IBM SPSS version 25.0 (IBM Co., Armonk, NY, USA) and JMP 14 (SAS Institute Inc., Cary, NC, USA).

## Results

**Fig. 1:**
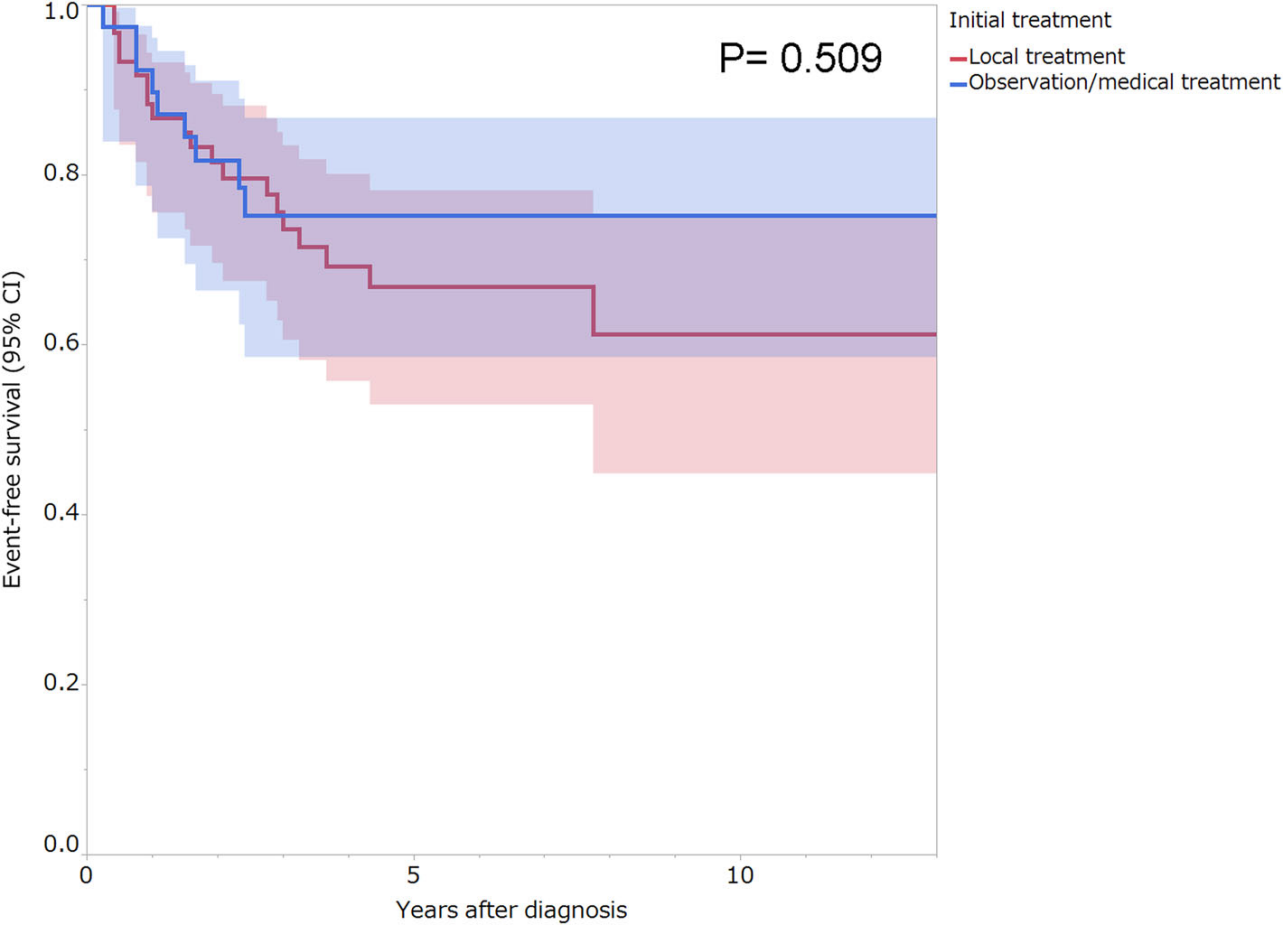
Kaplan-Meier analysis of event-free survival according to initial treatment. The shading surrounding the curves represents the 95% confidence interval

Overall, events (specific change in treatment strategy or recurrence) occurred in 28 patients (28.3%), and none of the patients died of disease. The univariate analysis revealed that male sex had a significant association with unfavorable event-free survival (*p* = 0.024; Table 3). No significant difference in event-free survival was observed between the first-line observation/medical treatment and local treatment groups (*p* = 0.509; Table 3, Fig. 1).

**Table 3:** Univariate predictors of event-free survival

Variable	Patients (n)	5-year event-free survival (95% CI, %)	***P*** value
***Age at diagnosis (years)***			
≤30	35	70.2 (52.8–83.3)	0.764
> 30	64	69.8 (56.3–80.6)	
***Sex***			
Male	37	56.3 (39.3–71.9)	0.024^a^
Female	62	77.8 (64.4–87.1)	
***Tumor site***			
Extremities	49	66.9 (51.8–79.1)	0.522
Non-extremities	50	73.1 (58.0–84.3)	
***Tumor size (cm)***			
≤5	25	82.8 (61.8–93.5)	0.345
5<, < 10	37	71.3 (54.2–83.9)	
≥10	37	60.1 (41.9–75.9)	
***Previous surgery or trauma at the site of the primary tumor***			
Yes	14	77.1 (47.9–92.5)	0.998
No	85	68.8 (57.4–78.3)	
***Year of diagnosis***			
1999–2009	61	64.9 (51.1–76.6)	0.372
2010–2018	38	78.8 (63.0–89.0)	
***Biopsy***			
Core needle biopsy	70	72.9 (60.4–82.6)	0.190
Open biopsy	29	63.2 (43.7–79.2)	
***Initial treatment***			
Local treatment	60	66.8 (53.0–78.2)	0.509
Observation/medical treatment	39	75.2 (58.6–86.7)	

^a^Statistically significant

First-line observation or medical treatment was more frequently chosen for larger tumors (*p* = 0.045) and between 2010 and 2018 (*p* < 0.001; Table 1). The number of surgical operations in the overall therapeutic process was higher in the first-line local treatment group than in the first-line observation or medical treatment group (*p* < 0.001; Table 2). The number of patients who received low-dose chemotherapy in the overall therapeutic process was significantly higher in the first-line observation or medical treatment group than in the first-line local treatment group (*p* < 0.001; Table 2). The median MSTS score of the 39 patients was 26 (IQR, 21–30). The median MSTS score of the 23 patients treated with first-line local treatment was 29 (IQR, 23–30), whereas that of the 16 patients managed with first-line observation or medical treatment was 21 (IQR, 19–29.5).

The univariate analysis for the patients initially treated with local treatment revealed that male sex was significantly associated with unfavorable recurrence-free survival (*p* = 0.021; Table 4). The surgical margins of the initial surgery in the surgery alone group were R0 (microscopic complete resection) in 23 patients, R1 (microscopic incomplete resection) in 16 patients, and R2 (macroscopic incomplete resection) in 6 patients. The surgical margins in the initial surgery in the “surgery with adjuvant radiotherapy” group were R0 in 6 patients, R1 in 8 patients, and R2 in 1 patient. The recurrence-free survival rates were not significantly different according to the surgical margin of the initial surgery and the presence of adjuvant radiotherapy in the univariate analysis (Table 4). The univariate analysis for the patients initially managed with observation or medical treatment revealed that none of the 8 variables (age, sex, tumor site, tumor size, previous surgery or trauma at the site of the primary tumor, year of diagnosis, biopsy, and initial treatment [observation+/−tamoxifen or NSAIDs vs. low-dose chemotherapy]) were significantly associated with event-free survival.

**Table 4:** Univariate predictors of recurrence-free survival in patients initially treated with local treatment

Variable	Patients (n)	5-year recurrence-free survival (95% CI, %)	***P*** value
***Age at diagnosis (years)***			
≤30	22	72.4 (50.6–87.1)	0.711
> 30	38	62.8 (44.6–78.0)	
***Sex***			
Male	23	50.2 (30.1–70.2)	0.021^a^
Female	37	76.9 (58.4–88.8)	
***Tumor site***			
Extremities	30	70.2 (50.4–84.6)	0.571
Non-extremities	30	63.0 (43.3–79.2)	
***Tumor size (cm)***			
≤5	17	86.9 (59.6–96.7)	0.295
5<, < 10	25	62.1 (41.5–79.1)	
≥10	18	55.2 (29.2–78.6)	
***Previous surgery or trauma at the site of the primary tumor***			
Yes	9	77.8 (42.1–94.4)	0.936
No	51	65.0 (49.9–91.7)	
***Year of diagnosis***			
1999–2009	48	65.1 (49.5–78.0)	0.750
2010–2018	12	75.0 (44.8–91.7)	
***Biopsy***			
Core needle biopsy	38	70.4 (52.8–83.5)	0.251
Open biopsy	22	60.6 (38.2–79.2)	
***Adjuvant radiotherapy***			
No	45	73.0 (57.2–84.5)	0.110
Yes	15	48.6 (23.8–74.1)	
***Surgical margin***			
R0	29	67.7 (48.4–82.4)	0.976
R1/2	31	66.4 (46.5–81.8)	

^a^Statistically significant. Macroscopic incomplete resection, R2; microscopic incomplete resection, R1; microscopic complete resection, R0

Among the patients who received low-dose chemotherapy in the overall therapeutic process, 3 (11.1%) experienced a partial response; 23 (85.1%), stable disease; and 1 (3.7%), disease progression. Among the patients treated with NSAIDs in the overall therapeutic process, 11 (64.7%) experienced stable disease and 6 (35.3%) experienced disease progression. The patient treated with a tyrosine kinase inhibitor exhibited a stable disease. Among the patients treated with combined NSAIDs and anti-hormonal therapy in the overall therapeutic process, 9 (75.0%) experienced stable disease and 3 (25.0%) displayed disease progression. Among the 16 patients who were managed with first-line observation, 7 (43.8%) experienced a disease progression; 2 (12.5%), stable disease; and 7 (43.8%), spontaneous regression.

## Discussion

Our results showed similar event-free survival between observation or medical treatment and local treatment as the first-line approach. The published studies regarding active surveillance for DT are summarized in Table 5 [3, 12–25]. Of patients under active surveillance, 4–37% spontaneously regressed, 17–92% had stable disease, and 4–59% had tumor progression [3, 12–25]. Kito et al. [26] performed a systematic review comparing surgery and active surveillance. They reported that the exacerbation rate (exacerbation; recurrence after surgery or progressive disease after active surveillance) was significantly higher in the surgery group (odds ratio, 1.32; 95% confidence interval [CI], 1.01–1.73; *p* = 0.05). Thus, our results confirmed that the use of first-line observation or medical treatment is appropriate for DT. A recent joint global consensus-based guideline from the Desmoid Tumor Working Group recommended that first-line treatment should start with a plan for active surveillance [5]. Indications for active treatment include pain, with or without radiological evidence of progression, functional symptoms, or patient request [27]. According to a recent systematic literature review of active surveillance for patients with DTs, the median follow-up time of the patients was reported in 12 studies and ranged from 8 to 73 months [28]. In the case of subsequent progression or a significant increase in symptom burden, a decision toward treatment may be considered and assessed with at least 3 further assessments and possibly not before 1 year after the initial diagnosis [29].

**Table 5:** Summary of the most important published studies on observation management of desmoid tumor patients

Study	Patients (n)	Abdominal/ Extra-abdominal (n)	Primary/Recurrent (n)	Median tumor size (cm)	Median follow-up (months)	Observation alone (n)	PD (n, %)	SD to PR (n, %)	Regression (n, %)	Tumor behavior	Comparison of PFS between observation and other treatments
Bonvalot et al. [12]	112	46/66	112/0	6	76	11	3 (27%)	NR	NR	NR	NR
Fiore et al. [3]	142	48/94	74/68	6	33	83	33 (40%)	47 (57%)	3 (4%)	47% PFS at 60 months	No difference in PFS between observation and chemotherapy
Barbier et al. [13]	26	0/26	11/15	NR	22	26	1 (4%)	24 (92%)	1 (4%)	Median time to stabilization 14 months	NR
Salas et al. [14]	426	120/306	426/0	7	52	27	6 (22%)	16 (59%)	5 (19%)	Median delay to progression 19.7 months	NR
Briand et al. [15]	73	0/73	31/24	7.5	73	55	5 (9%)	42 (76%)	5 (9%)	Median time to stabilization 9 months	NR
Huang et al. [16]	214	104/100	153/61	5.5	45	20	4 (20%)	14 (70%)	2 (10%)	Median delay to progression 15.3 months	NR
Park et al. [17]	47	7/40	47/0	8	36	20	1 (5%)	18 (90%)	1 (5%)	92% PFS at 36 months	Superior PFS in observation compared to surgery
Burtenshaw et al. [18]	213	213/0	195/18	6	54	63	4 (6%)	36 (57%)	23 (37%)	NR	NR
Penel et al. [19]	771	374/397	NR	5.7	32	388	117 (30%)	NR	NR	58% PFS at 24 months	No difference in PFS between observation and surgery
Orbach et al. [20]	154	NR	NR	NR	21	54	32 (59%)	9 (17%)	4 (7%)	NR	No difference in PFS between observation, surgery and chemotherapy
Cassidy et al. [21]	160	84/76	NR	4.7	15	37	10 (27%)	NR	NR	NR	NR
van Broekhoven et al. [22]	91	25/66	NR	NR	16	37	5 (14%)	25 (68%)	2 (5%)	NR	NR
van Houdt et al. [23]	168	76/92	NR	5.9	41	168	60 (36%)	60 (36%)	45 (27%)	NR	NR
Turner et al. [24]	103	42/61	NR	6.2	35	50	21 (42%)	29 (58%)	0	38% PFS at 36 months	NR
de Bruyns et al. [25]	227	89/138	NR	5.4	77	55	13 (24%)	20 (36%)	22 (40%)	71% PFS at 24 months	NR

*PD* progression disease, *SD* stable disease, *PR* partial response, *PFS* progression-free survival, *NR* not reported

This study showed poor extremity functional outcomes in the first-line observation/medical treatment group. However, larger tumors were more frequently managed with first-line observation or medical treatment, which may explain the reason for the poor extremity functional outcome in the first-line observation/medical treatment group. Newman et al. [6] reported that the Patient-reported Outcomes Measurement Information System function scores were lowest among patients who underwent ≥2 surgical interventions and among those treated with surgery and radiation at any time. Duhil de Benaze et al. [30] analyzed the long-term quality of life of pediatric patients with DT by using the Child Health Questionnaire. They did not find any difference in the quality-of-life scores between patients who underwent first-line observation and those who received more aggressive therapies (surgery or chemotherapy). Pain is not strictly correlated with DT progression; some stable DT may be painful, while some progressive DT may not be painful; sometimes, pain can be the consequence of previous locoregional treatments rather than the disease [31]. Therefore, unless the tumors are located in sites at risk of potentially life-threatening conditions, the most meaningful end point for evaluating the effectiveness of treatment strategies for DTs may be the impact on patient symptoms and function, rather than the traditional oncologic metrics such as local recurrence and disease progression on imaging [6, 23]. Thus, in the future, prospective data on function and quality of life must be collected from a larger cohort of patients with DTs.

In this study, observation or medical treatment was more frequently selected for larger tumors. Primary resection should be considered for small tumors, and medical therapy should be considered for large tumors [30]. Moreover, in this study, the patients treated with first-line local treatment needed less low-dose chemotherapy in their overall treatment than those managed with first-line observation or medical treatment. Duhil de Benaze et al. [30] and Sparber-Sauer et al. [32] reported the same results as ours. As first-line local treatments were more frequently selected for smaller tumors, low-dose chemotherapy might be less frequently indicated.

Our study shows that male sex had a significant association with unfavorable event- and recurrence-free survival. Most previous studies reported no association between sex and outcome [14, 16, 19, 21, 23, 33–35], while Huang et al. [36] reported that male sex was a predictor of local recurrence in the univariate analysis. Hong et al. [37] reported that testosterone regulates β-catenin protein levels and proliferation rates in DT. They found the possibility of therapeutic use of testosterone blockade in DT.

Our results showed that neither surgical margin nor adjuvant radiotherapy affected local recurrence. In 2017, Janssen et al. [38] performed a meta-analysis of the influence of surgical margins and adjuvant radiotherapy on local recurrence after resection of sporadic DT. In patients treated with surgical resection alone, the risk of local recurrence was almost twofold higher than that in patients with microscopically positive resection margins (risk ratio [RR], 1.78; 95% CI, 1.40–2.26) [38]. Adjuvant radiotherapy after surgery with negative margins had no detectable benefit on recurrence [38]. By contrast, after incomplete surgical resection, adjuvant radiotherapy improved the recurrence rates both in the patients with primary tumors (RR, 1.54; 95% CI, 1.05–2.27) and in those with recurrent DF (RR, 1.60; 95% CI, 1.12–2.28) [38]. Contrary to our results, evidence supports the effect of negative margins and adjuvant radiotherapy on local recurrence [38]. Turner et al. [24] suggested that negative margins should be the goal, but should not be pursued at the expense of significant functional loss. They suggested one additional consideration, namely tumor behavior [24]. Any residual from an aggressive tumor should be presumed to be at high risk of regrowth and that negative margins are therefore more important in this group [24]. On the other hand, more indolent disease may shift the balance in favor of minimizing surgical morbidity in cases that still require surgery [24].

Our study has several limitations. First, it was a retrospective study with inherent limitations and risk of selection bias. Smaller tumors were excluded from this study (Appendix). Second, the tumor size was not uniform among the first-line treatment groups. This may have affected the event-free survival or functional outcome. Third, a power analysis was not performed, and a risk of type II error due to the small sample size was present. If an adequate number of patients is gathered in the future, significant differences may appear regarding the other variables in this study. Forth, we were not able to compare the event-free survival between first-line observation and medical treatment groups due to the small sample size. Kasper et al. stated that a stepwise therapy escalation from an initial, less toxic treatment including observation to more toxic agents seems reasonable for progressive DTs [29]. These limitations should be considered in the analysis of our study results.

## Conclusions

Upfront surgery is not advantageous compared to more conservative treatments such as observation or medical treatment for patients with desmoid tumors.

## Supplementary Information

**Additional file 1: Appendix.** Patients characteristics of included or excluded patients in this study.

## Data Availability

The datasets generated, analyzed, or both during the present study are not publicly available because of privacy problems but are available from the corresponding author on reasonable request.

## Abbreviations

DT: Desmoid tumor
NSAID: Non-steroidal anti-inflammatory drug
IQR: Interquartile range
CT: Computed tomography
MRI: Magnetic resonance imaging
MSTS: Musculoskeletal Tumor Society
