# Supplementary material for: Upfront surgery is not advantageous compared to more conservative treatments such as observation or medical treatment for patients with desmoid tumors
Source: BMC Musculoskelet Disord. 2021 Jan 5;22:12. doi: 10.1186/s12891-020-03897-9 (PMC7784367; doi:10.1186/s12891-020-03897-9)
Supplement: Supplementary file 1 — Additional file 1: Appendix. Patients characteristics of included or excluded patients in this study. [file 12891_2020_3897_MOESM1_ESM.docx]

| **Appendix.** Patients characteristics of included or excluded patients in this study | | | | |
| --- | --- | --- | --- | --- |
| **Variables** | **Patients**  **(n= 253, %)** | **Included patients**  **(n= 99, 39.1%)** | **Excluded patients**  **(n= 154, 60.9%)** | **P value** |
| ***Age at diagnosis (years)*** | | | | |
| Median | 36 | 38.2 | 33 | 0.193 |
| IQR | 23.2-46.5 | 25.4-47.7 | 22.9-46 |  |
| ***Sex*** | | | | |
| Male | 92, 36.4% | 37, 37.4% | 55, 35.7% | 0.789 |
| Female | 161, 63.6% | 62, 62.6% | 99, 64.3% |  |
| ***Tumor site*** | | | | |
| Abdominal wall | 30, 11.9% | 4, 4.0% | 26, 16.9% | 0.662^a^ |
| Upper extremity | 30, 11.9% | 10, 10.1% | 20, 13.0% |  |
| Lower extremity | 89, 35.2% | 39, 39.4% | 50, 32.5% |  |
| Girdle | 61, 24.1% | 31, 31.3% | 30, 19.5% |  |
| Head and neck | 20, 7.9% | 6, 6.1% | 14, 9.1% |  |
| Chest wall | 19, 7.5% | 9, 9.1% | 10, 6.5% |  |
| Unknown | 4, 1.6% | 0 | 4, 2.6% |  |
| ***Tumor size (cm)*** | | | | |
| Median | 7 | 8 | 5 | 0.001* |
| IQR | 4.8-10 | 5-11 | 4-9 |  |
| ***Clinical presentation*** | | | | |
| None | 5, 2.0% | 0 | 5, 3.3% | 0.301^b^ |
| Mass | 103, 40.7% | 42, 42.4% | 61, 39.6% |  |
| Pain | 13, 5.1% | 7, 7.1% | 6, 3.9% |  |
| Mass + Pain | 100, 39.5% | 46, 46.5% | 54, 35.1% |  |
| Functional impairment | 4, 1.6% | 2, 2.0% | 2, 1.3% |  |
| Functional impairment + Pain | 8, 3.2% | 2, 2.0% | 6, 3.9% |  |
| Unknown | 20, 7.9% | 0 | 20, 13.0% |  |
| ***Previous surgery or trauma at the site of the primary tumor*** | | | | |
| Yes | 29, 11.5% | 14, 14.1% | 15, 9.7% | 0.451 |
| No | 208, 82.2% | 85, 85.9% | 123, 79.9% |  |
| Unknown | 16, 6.3% | 0 | 16, 10.4% |  |
| ***Year of diagnosis*** |  |  |  |  |
| 1978-2009 | 155, 61.3% | 61, 61.6% | 94, 61.0% | 0.711 |
| 2010-2018 | 91, 36.0% | 38, 38.4% | 53, 34.4% |  |
| Unknown | 7, 2.8% | 0 | 7, 4.6% |  |
| ***Biopsy*** |  |  |  |  |
| Core needle biopsy | 129, 51.0% | 70, 70.7% | 59, 38.3% | 0.462 |
| Open biopsy | 48, 19.0% | 29, 29.3% | 19, 12.3% |  |
| Unknown | 76, 30.0% | 0 | 76, 49.4% |  |
| ***Follow up period (months)*** |  |  |  |  |
| Median | 52 | 57 | 46.5 | 0.242 |
| IQR | 24-100.5 | 33-86 | 13.8-120.8 |  |
| IQR: interquartile range. *The difference was significant. ^a^Comparison of extremity and non-extremity. ^b^Comparison of none/mass alone and the others. | | | | |
